# Sero-epidemiological study of zoonotic bacterial abortifacient agents in small ruminants

**DOI:** 10.3389/fvets.2023.1195274

**Published:** 2023-07-28

**Authors:** Muhammad Abid Zeeshan, Sarmad Ali, Ishtiaq Ahmed, Aziz ur Rehman, Muhammad Kamran Rafique, Amar Nasir, Aman Ullah Khan, Muhammad Kashif, Katja Mertens-Scholz, Muhammad Imran Arshad, Syed Ehtisham-ul-Haque, Heinrich Neubauer

**Affiliations:** ^1^Department of Pathobiology (Pathology Section), University of Veterinary and Animal Sciences Lahore (Sub-Campus Jhang), Jhang, Pakistan; ^2^Department of Clinical Sciences, University of Veterinary and Animal Sciences Lahore (Sub-Campus Jhang), Jhang, Pakistan; ^3^Department of Pathobiology (Microbiology Section), University of Veterinary and Animal Sciences Lahore (Sub-Campus Jhang), Jhang, Pakistan; ^4^Institute of Bacterial Infections and Zoonoses, Friedrich-Loeffler-Institut, Jena, Germany; ^5^Institute of Microbiology, University of Agriculture, Faisalabad, Pakistan

**Keywords:** abortion, *Brucella*, *Coxiella*, *Chlamydia*, small ruminants

## Abstract

Abortion is one of the leading causes of economic losses in the livestock industry worldwide. *Chlamydia abortus*, *Coxiella burnetii*, and *Brucella* spp. are the leading cause of late fetal loss in small ruminants. This study determined the seroprevalence of these agents in small ruminants in district Jhang. A total of 385 serum samples were taken from the sheep and goats from different flocks with a history of abortion and subjected to i-ELISA. Further, samples were analysed for liver enzymes and total serum protein using a semi-automated chemistry analyzer. The result of indirect commercial ELISA showed 13.0, 4.2 and 11.2% prevalence for *Coxiella burnetii*, *Chlamydia abortus*, and *Brucella* spp., respectively. Values of different serum parameters (ALT, AST, and total protein) of seropositive animals were also determined. There was a significant rise in AST and ALT values of infected animals (*p* ≤ 0.05). Total protein decreased for all three infections, but a significant drop was noted in *Brucella* positive sheep serum samples. Various risk factors were studied. Binary logistic regression proved a significant role of ticks for coxiellosis and brucellosis. Age, parity, and species did not impact the prevalence of diseases studied.

## 1. Introduction

Abortifacient agents are the essential factors for tremendous losses in the livestock industry. These losses are caused by infertility, stillbirths, repeated breeding, decreased milk production, meat loss by aborted fetuses, additional treatment management costs, and veterinary services (1, 2). Moreover, abortion is a significant threat to public health when caused by zoonotic organisms. The most important zoonotic microorganisms causing abortions in small ruminants are *Brucella* spp., *Chlamydia abortus (C. abortus)*, and *Coxiella burnetii (C. burnetii)* (3).

Coxiellosis is caused by *C. burnetii*, an obligate intracellular, Gram-negative, and pleomorphic bacterium ranging in size from 0.4–1 μm in length and 0.1–0.4 μm in width. This agent has very similar characteristics to bacteria of the group Rickettsia (4). Once a neglected disease, it has attracted attention in human and veterinary medicine due to its zoonotic character and the economic losses caused. The organism can infect a wide range of animal species, but dairy animals, including cattle, buffaloes, sheep, and goats, are considered the main reservoirs of *C. burnetii* (5). It resides in the soil for a long period as it forms spore-like particles in harsh conditions and becomes resistant to high temperatures, ultraviolet radiation, and drying (6). Infection may occur after inhaling contaminated aerosols, uptake of contaminated feed, or ticks bites, while humans may also become infected after consuming contaminated raw milk food (5). Coxiellosis is usually an asymptomatic disease in many species. Clinical manifestations may involve abortion, stillbirth, premature delivery, and weak offspring in ruminants. Abortion in advanced pregnancy is the most important clinical observation of Q fever in sheep and goats (7). Q fever resembles atypical pneumonia in humans, a self-limiting flu-like disease that causes headaches, respiratory symptoms, and hepatitis (8). The chronic disease may result in endocarditis in humans (8, 9). The pathogen is shed in high numbers with secretions of infected animals into the environment at the time of parturition or abortion (10).

*Chlamydia abortus* causes abortion in sheep and goats worldwide and is known as Ovine Enzootic Abortion (OEA) or (EAE) Enzootic Abortion of Ewes (11, 12). *C. abortus* is a Gram-negative obligate intracellular bacterium. This disease causes acute placentitis and abortion in advanced pregnancy, particularly in the last 2–3 weeks. There may be noticed stillbirth or birth of weak lambs if abortion does not occur. Behavioral changes and vulvar discharge may be seen before abortion in some cases (13). Premature lambs often succumb within 24 h after birth (14). The vagina and placenta are covered with highly infectious pinkish and reddish-yellow colored exudates, respectively. Metritis, especially in goats, may also be found due to placenta retention due to secondary bacterial infection (15). Abortion, 2–3 weeks before the expected lambing time, could be the first clinical sign noticed. Uterine discharges contaminate the environment, and fetal fluids and organisms are shed with the infected placentae. Infection occurs via ingestion or inhalation of contaminated materials (16, 17).

Brucellosis in small ruminants is mainly caused by Gram-negative, non-motile, highly contagious, zoonotic bacteria of *Brucella melitensis*. High economic losses result from sterility, fetal death, or abortion (7). The pathogen is shed in large numbers in feces, urine, milk, and reproductive secretions by infected animals, spreading the disease to other animals and humans and posing a considerable threat to public health. The animals may get infected by ingesting or inhaling the contaminated feed and air. Wounds, bruises, or any discontinuity of the skin can also be a point of entry for the bacteria (18).

Abortifacient bacteria cause serious health problems in small ruminants by damaging vital organs such as the liver, kidney, and heart (19). The injury to these organs results in biochemical alterations, for instance, the necrosis of hepatocytes due to *Brucella* spp. results in the increase of certain liver enzymes such as ALT and AST and a decrease in proteins produced by the liver (20, 21); similarly, a study conducted in Iraq reported the biochemical changes due to chlamydiosis in ewes (22). However, the data is scanty about the biochemical alteration caused by abortifacient bacteria in small ruminants and needs further investigation.

In the current study, we investigated the prevalence of antibodies against *C. abortus*, *C. burnetii*, and *Brucella* spp. and identified risk factors along with the effects of these infections on liver enzymes and total serum protein.

## 2. Materials and methods

The study was conducted in District Jhang. Ethical approval was taken from the Ethical Review Committee (IRC) of UVAS. The study area comprises four tehsils (Jhang, Athara Hazari, Shorkot, and Ahmadpur Sial). The sample size essentially required to carry out the present study was determined by using the formula *n* = 1.96^2^ 𝑃exp (1 − *P*exp)/𝑑^2^ with a 95% confidence interval, where *n* = required sample size, *P* = expected prevalence, and *d* = desired absolute precision (23). A prevalence of 50% was expected. A total of 385 serum samples were collected from animals with a history of abortion within the last month (30 days) randomly from all over the district. During sampling, data of the individual animals, i.e., species, parity, age, and presence of ticks, were collected using a questionnaire. The samples were collected during winter, from November to March, as most parturitions and abortions in small ruminants occur in these months. Out of 385 serum samples, 235 samples were taken from goats, and 150 samples were taken from sheep depending upon the availability of aborted animals of both species not previously vaccinated. Animals were divided into two age groups, 1–3 years and 3–6 years. Whole blood was collected from the jugular vein of sheep and goats with a history of abortion using gel clot vacutainers. After clotting at room temperature, the samples were centrifuged at 1,500 rpm for 10 min. Supernatants were stored in 1.5 mL aliquots at −80°C until use.

### 2.1. Serological analysis

Antibodies against *Brucella* spp. were detected using ID Screen® Brucellosis Serum Indirect Multi-species ELISA (IDvet, France) (24) and against *C. burnetii* using an indirect Enzyme-Linked -Immunosorbent Assay (ID Screen® Q Fever Indirect Multi-species, IDvet, France) (25). This kit is based on phase I and II *C. burnetii* antigens.

The serum antibodies against *C. abortus* were detected using the commercial ELISA kit (ID Screen® *Chlamydophila abortus* Indirect Multi-species, CHLMS-MS-2P / CHLMS-MS-5P) (26). The ID Screen *C. abortus* Indirect ELISA uses a synthetic antigen from a major outer-membrane protein (Momp) specific to *C. abortus*, which reduces the frequency of non-specific reactions. The kits were used according to the instructions of the manufacturers.

### 2.2. Biochemical analysis

A total of 86 serum samples from aborted (39 goats and 27 sheep) and clinically healthy animals (10 goats and 10 sheep) were analyzed for serum biochemistry (27). Total protein (TP) and serum enzymes ALT and AST were estimated on an automated chemistry analyzer (Optizen HF 1412, Korea) using commercial kits (Bioactiva diagnostica GmbH, Germany) according to the manufacturer’s recommendations (28).

### 2.3. Statistical analysis

Data regarding antibody seroprevalence was analyzed using the chi-square test, while the impact of different risk factors, such as area, age, parity, etc., was estimated by binary logistic regression. Variations in the concentrations of serum enzymes and total protein were analyzed by one way ANOVA test. A value of *p* lower than 0.05 was considered significant.

## 3. Results

### 3.1. Seroprevalence of anti-*Coxiella burnetii*, *Chlamydia abortus* and *Brucella* spp. antibodies in sera of sheep and goats

The serological analysis revealed a comparable prevalence of antibodies against the three abortifacient agents, *C. burnetii*, *C. abortus*, and *Brucella* spp., in sheep and goats. No significant difference was found between the prevalence of these pathogens in sheep and goats (Table 1).

**Table 1:** Prevalence and anti-*Coxiella burnetii*, *Chlamydia abortus* and *Brucella* spp. antibodies in sheep and goats of Jhang, Pakistan.

Parameter		*Coxiella burnetii*			*Chlamydia abortus*			*Brucella* spp.		
		+ve (%)	Total	*p* value	+ve (%)	Total	*p* value	+ve (%)	Total	*p* value
Species	Sheep	19 (12.6)	150	0.38	5 (3.3)	150	0.6	24 (16)	150	0.19
	Goats	31 (13.1)	235		11 (4.6)	235		19 (8)	235	
	Total	50 (13.0)	385		16 (4.2)	385		43 (11.2)	385	
Parity	1st–3rd	29 (16.7)	174	0.124	7 (4.0)	174	0.5	15 (8.6)	174	0.45
	4th–6th	21 (10.0)	211		9 (4.3)	211		28 (13.2)	211	
	Total	50 (13.0)	385		16 (4.2)	385		43 (11.2)	385	
Age (years)	1–3	33 (15.1)	218	0.3	9 (4.1)	218	0.5	30 (13.8)	218	0.5
	4–6	17 (10.2)	167		7 (4.2)	167		13 (7.8)	167	
	Total	50 (13.0)	385		16 (4.2)	385		43 (11.2)	385	

The values in the parenthesis are indicating percentile prevalence.

**Figure 1:**
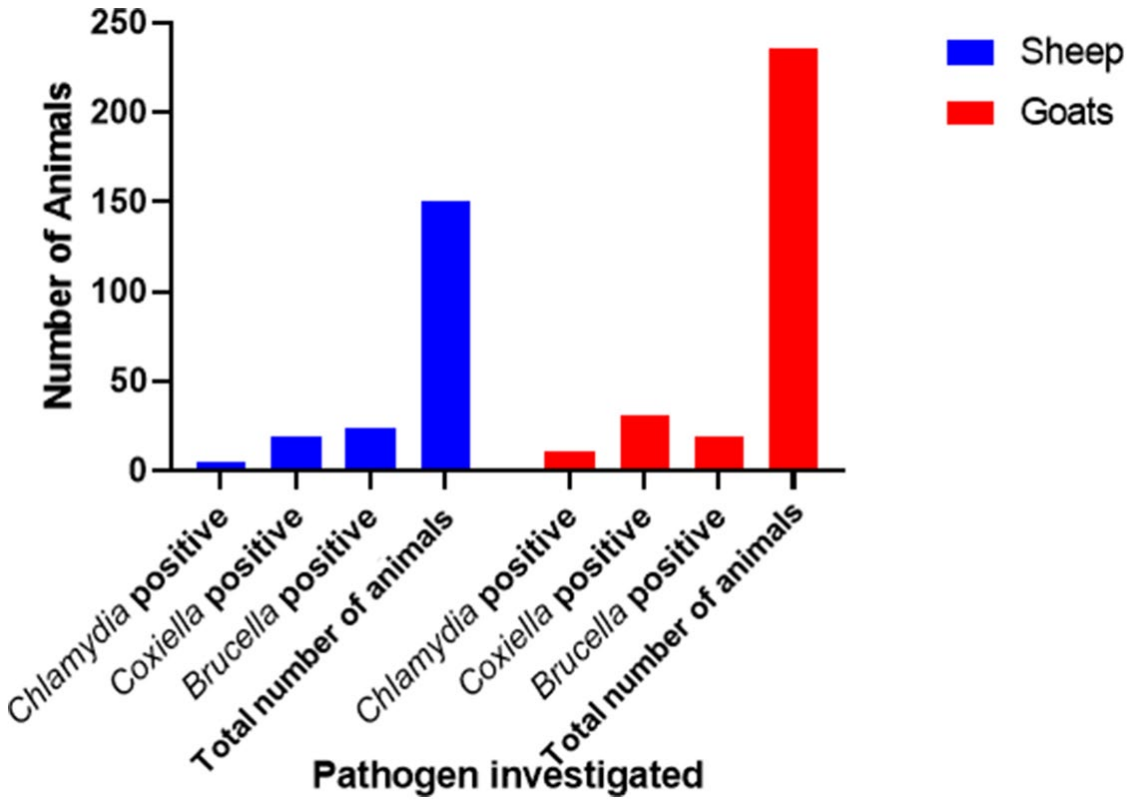
Prevalence of anti-*Chlamydia, Coxiella*, and *Brucella* spp. antibodies in small ruminants in Jhang, Pakistan.

There is a total prevalence of 13.0% for anti-*Coxiella burnetii*, 4.2% for anti-*Chlamydia abortus*, and 11.2% for anti-*Brucella* spp. antibodies were found in small ruminants. Risk factors like parity and age of animals showed a no-significant relationship with the seroprevalence. Anti-*Coxiella burnetii* antibodies’ prevalence was 12.6% in sheep and 13.1% in goats (Figure 1). In the case of anti-*Chlamydia abortus* antibodies, 3.3% prevalence was found in sheep and 4.6% in goats. The prevalence of anti-*Brucella* spp. antibodies were 16% in sheep and 8% in goats. No statistically significant relation was found for host prevalence or study area.

### 3.2. Risk factors

**Figure 2:**
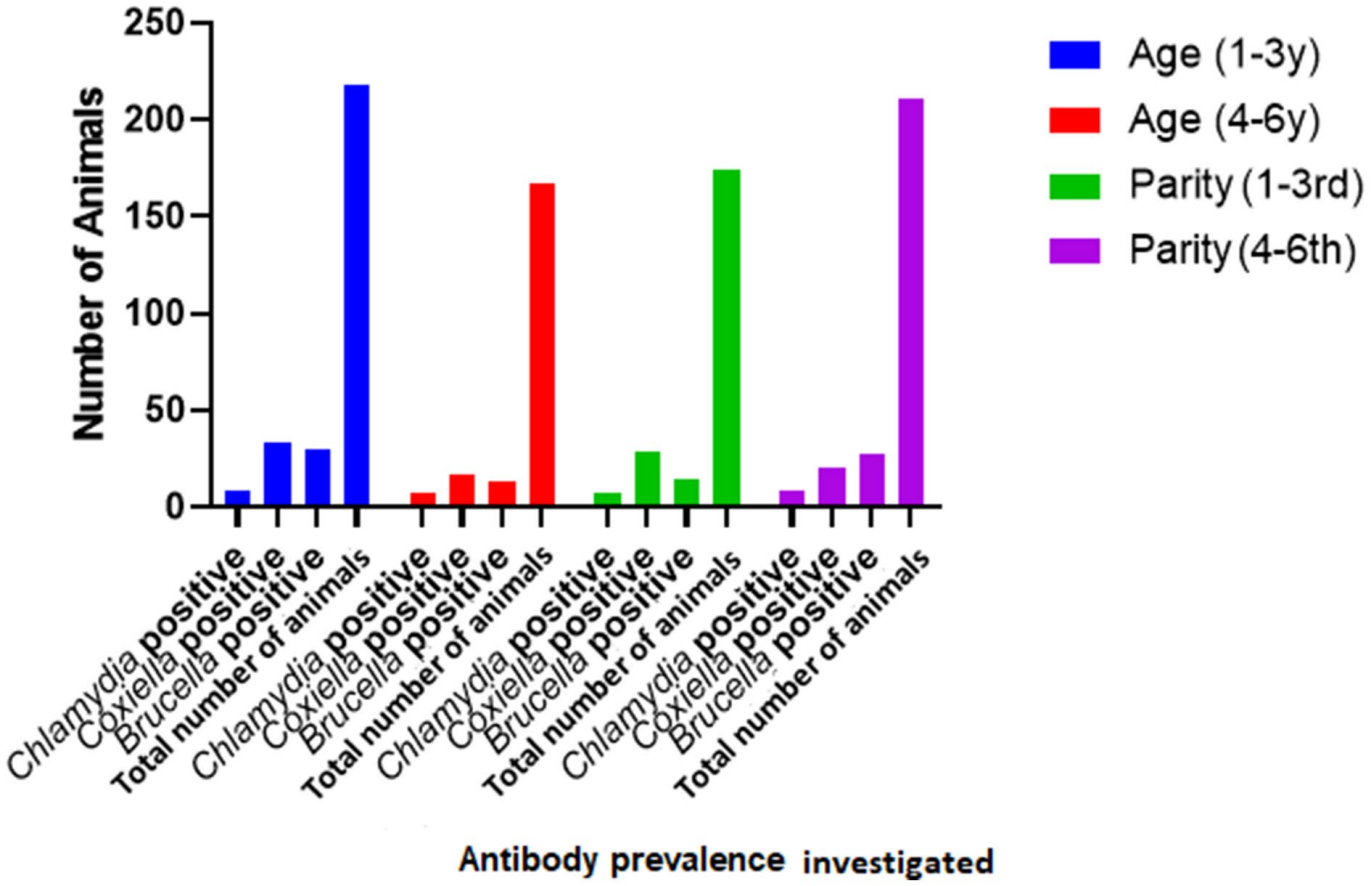
Relationship of seroprevalence with age and parity.

Species of animals (sheep and goats), age (two age groups: 1–3 years and 3–4 years), and parity were studied as possible risk factors. Statistical analysis (binary logistic regression) revealed that the species, age, and parity have no significant impact as risk factors (Figure 2). However, ticks play a significant role in the occurrence of anti-*C. burnetii* antibodies. Statistical analysis showed no significant relationship between anti-*Brucella* antibodies with ticks. The results are described in Table 2.

**Table 2:** Binary logistic regression statistics of risk factors.

Risk factor	95% C-I	Odd ratio	value of *p*
**Anti*-Coxiella burnetii* antibodies**			
Species	0.65–1.64	1.431	0.373
Age	0.30–2.41	0.863	0.778
Parity	0.19–1.39	0.518	0.194
Ticks	3.97–21.3	9.213	0.000
**Anti*-Chlamydia abortus* antibodies**			
Species	0.743–6.640	2.221	0.153
Age	0.239–4.132	0.995	0.994
Parity	0.291–5.11	1.220	0.784
**Anti-*Brucella* antibodies**			
Species	0.205–3.858	0.889	0.876
Age	0.027–3.078	0.288	0.303
Parity	0.151–11.282	1.306	0.808
Ticks	0.657–13.189	2.945	0.157

### 3.3. Serum biochemistry

The trends for increased or decreased serum enzymes (ALT and AST) and total protein with the prevalence of antibodies against the investigated pathogens were similar. A significant increase in ALT and AST was noted, while the total serum protein concentration was decreased. Detailed statistics are shown in Tables 3–5.

**Table 3:** Relations of anti *C. abortus, C. burnetii* and *Brucella* spp. antibodies prevalence to serum enzyme ALT and AST in sheep.

	ALT			AST	
	Number	Mean ± S.D	Value of *p*	Mean ± S.D	Value of *p*
*Chlamydia* positive	10	42.3 ± 9.9^b^	0.000	134.1 ± 46.0^b^	0.00
*Coxiella* positive	7	49.1 ± 4.7^b^	0.000	100.8 ± 12.6^a^	0.88
*Brucella* spp. positive	10	57.4 ± 7.3^b^	0.001	90.2 ± 12.6^a^	0.33
Healthy animals	10	29.5 ± 5.7^a^		59.5 ± 9.0^a^	

The values of ALT and AST with different superscripts in each column show significant difference among infected and healthy animals.

**Table 4:** Relations of anti *C. abortus, C. burnetii* and *Brucella* spp. antibodies prevalence to ALT and AST in Goats.

	ALT			AST	
	Number	Mean ± S.D	Value of *p*	Mean ± S.D	Value of *p*
*Chlamydia* positive	11	40.0 ± 8.9^b^	0.003	92.54 ± 3^b^	0.001
*Coxiella* positive	18	45.6 ± 9.7^b^	0.001	87.83 ± 2^b^	0.002
*Brucella* spp. positive	10	69.4 ± 6.3^b^	0.000	100.80 ± 8^b^	0.001
Healthy animals	10	10.9 ± 1.7^a^		64 ± .2^a^	

The values of ALT and AST with different superscripts in each column show significant difference among infected and healthy animals.

**Table 5:** Relations of anti *C. abortus, C. burnetii* and *Brucella* spp. antibodies prevalence to total protein in sheep and goats.

	Sheep				Goat			
	Number	Mean ± S.D	CI 95%	Value of *p*	Number	Mean ± S.D	CI 95%	Value of *p*
*Chlamydia* positive	10	5.46 ± 1.54^a^	4.35–6.56	0.946	11	6.0 ± 1.0^a^	4.14–8.6	0.50
*Coxiella* positive	7	4.67 ± 0.72^a^	3.9–5.34	0.284	18	6.6 ± .84^a^	5.10–8.6	0.20
*Brucella* spp. positive	10	4.07 ± 1.79^b^	2.7–5.35	0.027	10	4.4 ± .55^a^	3.0–5.5	0.92
Healthy animals	10	5.73 ± 0.87^a^	5.1–6.35		10	4.9 ± .31^a^	4.21–5.6	

The values of total protein with different superscripts in each column show significant difference among infected and healthy animals.

## 4. Discussion

Coxiellosis, brucellosis, and chlamydial infections are diseases of zoonotic importance and impose devastating economic losses to the livestock industry by decreased production and reduced reproduction (29, 30). Early diagnosis of diseases has a positive effect on the successful treatment of animals and may prevent the spread of disease to other animals and human beings (31, 32). Further, the economic losses to the livestock industry and costs for the public health sector may be prevented (33, 34). In Pakistan, data on the geographic distribution of Q fever based on epidemiological surveillance studies are limited for livestock and humans. In the current research, the prevalence of *C. burnetii* infection, i.e., the seroprevalence in small ruminants, has been investigated using indirect ELISA, the serodiagnostic technique is preferably used for screening as it is more sensitive and specific than any other serological technique (35–37) reported 100% sensitivity and specificity for the applied IDVET® Q fever Indirect ELISA. Thus, 13.0% sero-epidemiology of the pathogen in district Jhang is a significant finding concerning future disease management in animals and humans.

The current study investigated the relationship between the risk factor age with anti-*Coxiella burnetii* antibody prevalence in small ruminants. The statistical analysis revealed that age is not a significant risk factor. This result coincides with the results of the previous study (37). It can be assumed that the way of keeping the herds (mixed age group and species) results in chronic infection of the herd and infection pressure on the individual animal independent of its age. There was no significant difference concerning the prevalence of coxiellosis found among different four tehsils of District Jhang. This indicates that the disease is prevalent and endemic in District Jhang. Our study shows no significant difference in the prevalence of coxiellosis in sheep and goats, indicating that both species are equally susceptible to the disease and share the same epidemiological environment. These results are similar to a previous study conducted in Punjab, Pakistan (18). Notably, ewes and goats are more prevalent during the first, second, and third pregnancy than those with higher parities. The relationship between the prevalence of disease with the parity of animals has also been evaluated in the current study. Animals with the first three parities indicated a higher disease prevalence than those with higher parities (38). It can be assumed that older females may have developed a certain degree of immunity that hinders re-infection.

Biochemical analysis indicated the stoking rise of the amount of the ALT and AST serum enzymes. This may be due to the chronic infection, e.g., ongoing inflammation of placental tissue. Chronic infection of the liver by *C. burnetii* is also reported by various studies. This chronic infection is responsible for the increase of ALT and AST and the decrease in the total protein concentration in the serum. Our results coincide with the findings of (39). There are several reports of hepatitis associated with *C. burnetii* infection in humans as well (40, 41).

Our study also studied different risk factors for infection with *C. abortus*, such as species, age, and parties. A higher sero-prevalence was recorded in goats when compared to sheep. Five out of 150 sheep (3.3%), while 11 out of 235 caprine samples tested positive (4.6%). This higher prevalence in goats may be a sampling bias. However, statistical analysis showed that the host species is indeed no significant risk factor (*p* > 0.05), as already described in a recent study (42). Though statistically non-siginifcant (*p* > 0.05), however, it has been found that animals of the 1-3 year age group were more often found positive than those of the age group older than 3 years. This finding agrees with the findings of a previous study (43). Possible reasons for these findings have already been described above.

Brucellosis is also prevalent in Pakistan, causing significant losses for animal owners and posing a severe risk to human health (18). Serology is the preferred choice for diagnosing brucellosis to reduce the high risk of infection for laboratory personnel during cultivation (44). ELISA offers easy handling, high sensitivity and specificity. Our study revealed 11.2% prevalence in small ruminants from District Jhang. Serum parameters such as AST, ALT, and total protein were studied in infected animals. There was a significant increase in the values of AST and ALT in the infected animals. Mean values of AST and ALT were higher in the animals infected with *Brucella*. These results are in agreement with a previous study (45). The reasons for our findings have been discussed already.

Hence blood parameters need further investigation. In this study, ALT and AST values were increased in sheep and goats, as found in a previous study (22). In contrast, Kushwaha et al. (46) found a significant decrease in ALT and AST. The total protein showed a non-significant decrease in sheep and goats. The decrease in total protein may be caused by the damage of the endothelium of the liver due to chronic intracellular infection leading to decreased production of liver proteins (47). The excretion of proteins in urine may be an indicator of renal infection. Chronic liver infection may also cause extensive damage to the liver cells, which release ALT. Consequently, an increase in ALT activity is measured in the serum (48). Although generally thought to be specific to the liver, ALT is also found in the kidneys and, in much smaller quantities, in heart and skeletal muscle cells. In acute hepatocellular injury, serum AST levels usually rise immediately, reaching higher levels than ALT within 24–48 h. If ongoing chronic damage occurs, ALT levels will become higher than AST levels because of their higher plasma half-life. Finally, ALT levels are more commonly elevated than AST levels (49). In human medicine, elevated levels of serum liver enzymes have been observed in hepatitis due to brucellosis (50).

## 5. Conclusion

Brucellosis, and coxiellosis have reltively higher prevalence than Chalmydiosis in the study area. This is the first study reporting the prevalence of *C. abortus* in small ruminants in Punjab province of Pakistan. This study further concludes that ticks are important risk factors for the occurance of coxiellosis and brucellosis. These infections may also affect the liver function as indicated by a rise in the serum levels of liver enzymes and decrease in the total protein concentration. Additional studies are required to estimate the prevalence of *C. abortus* in small ruminants in other areas of the country.

## Data availability statement

The original contributions presented in the study are included in the article/supplementary material, further inquiries can be directed to the corresponding authors.

## Ethics statement

The animal study was reviewed and approved by Ethical Review Committee, University of Veterinary and Animal Sciences Lahore (Sub Campus Jhang). Written informed consent was obtained from the owners for the participation of their animals in this study.

## Author contributions

MZ, SA, IA, and AR planned and executed the study. MR, AN, MK, and AK contributed in the methodology and data analysis. KM-S, MA, SE-u-H, and HN performed data analysis, drafting and editing manuscript. All authors contributed to the article and approved the submitted version.

## Funding

This research was supported by the International Foundation for Science (IFS), Stockholm, Sweden, through a grant (no. I-3-B-6346-1) to Ishtiaq Ahmed, Associate Professor at the University of Veterinary and Animal Sciences Lahore (Jhang Campus).

## Conflict of interest

The authors declare that the research was conducted in the absence of any commercial or financial relationships that could be construed as a potential conflict of interest.

## Publisher’s note

All claims expressed in this article are solely those of the authors and do not necessarily represent those of their affiliated organizations, or those of the publisher, the editors and the reviewers. Any product that may be evaluated in this article, or claim that may be made by its manufacturer, is not guaranteed or endorsed by the publisher.
