# Corrigendum: Sero-epidemiological study of zoonotic bacterial abortifacient agents in small ruminants

**DOI:** 10.3389/fvets.2023.1287165

**Published:** 2023-09-14

**Authors:** Muhammad Abid Zeeshan, Sarmad Ali, Ishtiaq Ahmed, Aziz ur Rehman, Muhammad Kamran Rafique, Amar Nasir, Aman Ullah Khan, Muhammad Kashif, Katja Mertens-Scholz, Muhammad Imran Arshad, Syed Ehtisham-ul-Haque, Heinrich Neubauer

**Affiliations:** ^1^Department of Pathobiology (Pathology Section), University of Veterinary and Animal Sciences Lahore (Sub-Campus Jhang), Jhang, Pakistan; ^2^Department of Clinical Sciences, University of Veterinary and Animal Sciences Lahore (Sub-Campus Jhang), Jhang, Pakistan; ^3^Department of Pathobiology (Microbiology Section), University of Veterinary and Animal Sciences Lahore (Sub-Campus Jhang), Jhang, Pakistan; ^4^Institute of Bacterial Infections and Zoonoses, Friedrich-Loeffler-Institut, Jena, Germany; ^5^Institute of Microbiology, University of Agriculture, Faisalabad, Pakistan

**Keywords:** abortion, *Brucella*, *Coxiella*, *Chlamydia*, small ruminants

In the published article, an author name was incorrectly written as Katja Mertns-Scholz. The correct spelling is Katja Mertens-Scholz.

The authors apologize for this error and state that this does not change the scientific conclusions of the article in any way. The original article has been updated.

